# Evaluation of 0.2 µg/day fluocinolone acetonide (ILUVIEN) implant in a cohort of previously treated patients with diabetic macular oedema (DMO): a 36-month follow-up clinical case series

**DOI:** 10.1136/bmjophth-2020-000484

**Published:** 2020-07-05

**Authors:** Muna Ahmed, Christine Putri, Hibba Quhill, Fahd Quhill

**Affiliations:** Ophthalmology, Sheffield Teaching Hospitals NHS Foundation Trust, Sheffield, Yorkshire, UK

**Keywords:** macula, retina, inflammation, vision, treatment medical

## Abstract

**Objective:**

To assess the real-world effectiveness and safety of single injection of a fluocinolone acetonide (FAc) implant in previously treated patients with recurrent diabetic macular oedema (DMO) over a 36-month follow-up period.

**Methods and Analysis:**

This is a retrospective study conducted at a single ophthalmology department at the Royal Hallamshire Hospital, Sheffield, UK. Data were collected using electronic medical records to identify all patients treated with a FAc implant for DMO between March 2014 and November 2014, followed with a 36-month clinic follow-up. Outcomes measured included mean change in best-recorded visual acuity (BRVA) and central macular thickness (CMT) over the period of 36 months, treatment burden pre-implant and post-implant, and functional and anatomical responder rates.

**Results:**

Twenty-six eyes (n=22 patients) were treated with single intravitreal FAc implant followed with 36 months of follow-up. At 24 and 36 months, 86.4% and 75.0% of patients maintained or gained vision post-FAc implant in routine clinical practice. The mean BRVA increased from 41.8 to 54.6 letters at month 24 and 45.8 letters at month 36, with 50.0% and 33.3% of patients achieving a ≥15 letter improvement at months 24 and 36, respectively. The mean CMT reduced from 600.8 µm at baseline to 351.0 µm and 392.5 µm at months 24 and 36, respectively. Overall, a mean of one treatment every 13.33 months post-FAc implant (vs 3.24 months pre-FAc implant) was reported. Eleven eyes had an increased intraocular pressure of ≥10 mm Hg and 12 eyes had an increase to ≥25 mm Hg from baseline.

**Conclusion:**

These results further support the effectiveness and safety of FAc implant in previously treated patients with persistent or recurrent DMO in a real-world clinical practice.

**Key messages**

**What is already known about this subject?**

- The pivotal trials (Fluocinolone Acetonide in Diabetic Macular Edema A and B) followed patients for 3 years and showed the efficacy and safety of the fluocinolone acetonide (FAc) implant in a controlled setting.
- How the FAc implant performs in real-world practice is unknown as there are limited studies documenting its long-term effectiveness in current practice.

**What are the new findings?**

- A single FAc implant led to structural and functional improvements that lasted 3 years.
- Benefits included the stabilisation of visual acuity and a sustained reduction in retinal oedema, which were associated with a marked reduction in treatment burden (ie, fewer intravitreal treatments), in which additional treatment was required every 13.33 months as opposed to every 3.24 months pre-FAc implant.
- The clinical impact was a reduction in the number of hospital visits over 3 years, from a total of 570 visits prior to FAc implant to 462 visits post-FAc implant.

**How might these results change the focus of research or clinical practice?**

- Sustained therapeutic benefits combined with fewer supplemental treatment injections and hospital visits help to reduce medication burden in patients with diabetic macular oedema and at an increased risk of eyesight loss.
- The FAc therapy may also help to reduce clinical appointment burden and help to make clinic visits more efficient and effective.

## Introduction

Diabetic macular oedema (DMO) is the most common visual complication of diabetes and diabetic retinopathy (DR) in working-age adults.^1^ The prevalence of diabetes for all age groups worldwide is estimated to rise to 333 million by 2025.^2^ The global prevalence of DR among individuals with diabetes is estimated to be 35%, with DMO present in 6.8%.^3^

Current treatment options include laser photocoagulation, intravitreal antivascular endothelial growth factor (anti-VEGF) injections and intravitreal corticosteroid therapies.^4–8^ It is estimated that between 31.6% and 65.6% of patients with DMO respond suboptimally to anti-VEGFs.^9^ Furthermore, patients who do not respond well after the first three loading injections are unlikely to respond well in the long-term with anti-VEGF monotherapy, and therefore a change in therapy should be considered.^10^

Visual acuity (VA) improvement with long-term anti-VEGF therapy (ranibizumab) appeared to be negatively associated with residual, persistent DMO.^11^ This highlights the need for alternative, ideally more effective, treatment strategies for patients with DMO that respond insufficiently to intermittent therapies.^9^

Intravitreal corticosteroid implants are effective for the treatment of DMO, with sustained-release drug delivery (such as OZURDEX and ILUVIEN) reducing disease reoccurrences. These treatments also reduce the number of injections and follow-up visits compared with anti-VEGFs, thus helping to improve patient compliance and lowering disease burden.^5–7 10 12^

ILUVIEN (0.19 mg of fluocinolone acetonide (FAc)) is a non-biodegradable intravitreal implant that releases 0.2 µg/day of FAc for up to 36 months.^12^ The FAc implant is indicated for the treatment of vision impairment associated with chronic DMO, considered insufficiently responsive to available therapies (ie, persistent or recurrent DMO despite treatment).^13^ The efficacy and safety profile of the FAc implant in DMO was demonstrated in two phase III Fluocinolone Acetonide in Diabetic Macular Edema (FAME) trials.^5 6^

The National Institute for Health and Care Excellence (NICE) recommends the use of the FAc implant as an option for treating chronic DMO that is insufficiently responsive to available therapies only if the implant is to be used in an eye with an intraocular (pseudophakic) lens.^14^ There is a paucity of real-world data following treatment with FAc implants and studies can be criticised for limited follow-up.^15–18^

The primary objective of this retrospective case series was to assess the effectiveness, safety and treatment burden in a cohort of patients with DMO that persisted or recurred despite treatment and that were treated with a single FAc implant over a 36-month follow-up period.

## Materials and methods

### Study design

This study involved retrospective data collection using a single electronic medical records system (Medisoft Ophthalmology, Medisoft, Leeds, UK), which was used to identify patients treated with a 0.2 µg/day FAc implant. Patients were treated between March 2014 and November 2014 and followed up for 36 months. Data from patients were included if they had received the 0.2 µg/day FAc implant for the treatment of chronic DMO.

The study was conducted at a single site in the ophthalmology department of the Royal Hallamshire Hospital, Sheffield, UK. Prior to FAc implant, an 18-month period was defined (this allowed prior therapies to be collected over the period when anti-VEGF and corticosteroid intravitreal therapies had been approved for use in DMO), so that DMO therapies in this period could be standardised by time and compared with the 36-month period post-FAc implant administration. Treatment was carried out according to clinicians’ discretion.

### Data extraction and analysis

Patient baseline demographics were recorded, including age, sex, gender, duration of diabetes, type of diabetes, haemoglobin A1c (HbA1c), lens status and prior treatments. Ocular characteristics, such as best-recorded visual acuity (BRVA), were recorded for each eye, at baseline and throughout the 36-month period, using metric notation from the Snellen chart; Snellen VA fractions were converted to approximate Early Treatment Diabetic Retinopathy Study (ETDRS) letter scores. Central macular thickness (CMT; recorded using Heidelberg Spectralis with all patients undergoing macular scans using the same system and Ocular Coherence Tomography (OCT) protocol) and intraocular pressure (IOP)-related events were also recorded.

Study parameters included mean changes in BRVA and CMT from baseline; VA stabilisation (defined as a change of ±4 ETDRS letters from baseline); VA improvement (an increase of ≥5 ETDRS letters from baseline); anatomical and functional responses (with functional and anatomical responses based on a ≥5 letter gain and a ≥20% CMT reduction, respectively)^19 20^; treatment burden pre-FAc and post-FAc implant; and, the total number of eye clinic visits pre-FAc and post-FAc implant. Measurements were conducted at the following time points: −36, −30, −24, −18, −12, −6, −3, 0, first follow-up (within 1 month), 3, 6, 12, 18, 24, 30 and 36 months. Last observation carried forward was also calculated to account for two cases where data were collected to month 30.

### Patient involvement

Patients were not involved in the design of this study.

### Data and statistical analyses

Data were extracted from the entire record of eligible patients so that observations and treatments before and after 0.2 µg/day FAc treatment were included. All comparisons were descriptive, except for (1) mean changes in BRVA and CMT from baseline to months 6, 24 and 36; and, (2) the total number of hospital visits in the 36 months pre-FAc and post-FAc administration. A Student’s paired t-test was used to compare these time points, and a statistical difference was taken as p≤0.05.

## Results

### Patient population

Overall, 26 eyes of patients (n=22) with recurrent or persistent DMO were identified and were treated for 36 months with an FAc intravitreal implant (4 patients received bilateral treatment).

The baseline demographics and ocular characteristics are summarised in table 1. The mean age of patients was 68.4 years; 80.8% of patients had type 2 diabetes and the mean duration of diabetes was 20.3 years. All patients were pseudophakic at baseline and were treated according to NICE guidance.^13^ The mean baseline BRVA was 41.8 ETDRS letters and the mean CMT was 600.8 µm. In terms of prior treatment, majority of the eyes (n=25, 96.2%) received intravitreal therapy prior to FAc implant.

**Table 1:** Baseline demographics and ocular characteristics

Baseline characteristics	Eyes (n=26 from 22 patients)
Age, years (mean±SD)	68.4±10.2
Gender, male/female, n	16/6
Diabetes duration, years (mean±SD)	20.3±11.8
Type of diabetes, n (%)	
Type 2	21 (80.8)
Type 1	5 (19.2)
HbA1c, mmol/mol (mean±SD)	64.5±18.4
Pseudophakic, n (%)	26 (100)
BRVA, ETDRS letters (mean±SD)	41.8±19.2
CMT, µm (mean±SD)	600.8±160.4
Prior intravitreal therapies (mean±SD), n (%)	7.7±3.8, 25 (96.2)
Anti-VEGF (mean±SD), n (%)	7.3±3.5, 21 (80.0)
Any intravitreal corticosteroid (mean±SD), n (%)	1.9±1.2, 20 (76.9)
Macular laser (mean±SD), n (%)	1.4±0.5, 7 (26.9)
PRP (mean±SD), n (%)	1.8±0.8, 5 (19.2)

HbA1c; haemoglobin A1c; BRVA, best-recorded visual acuity; CMT, central macular thickness; ETDRS, Early Treatment Diabetic Retinopathy Study; PRP, panretinal photocoagulation; VEGF, vascular endothelial growth factor.

### Effectiveness assessments

#### Best-recorded visual acuity

**Figure 1:**
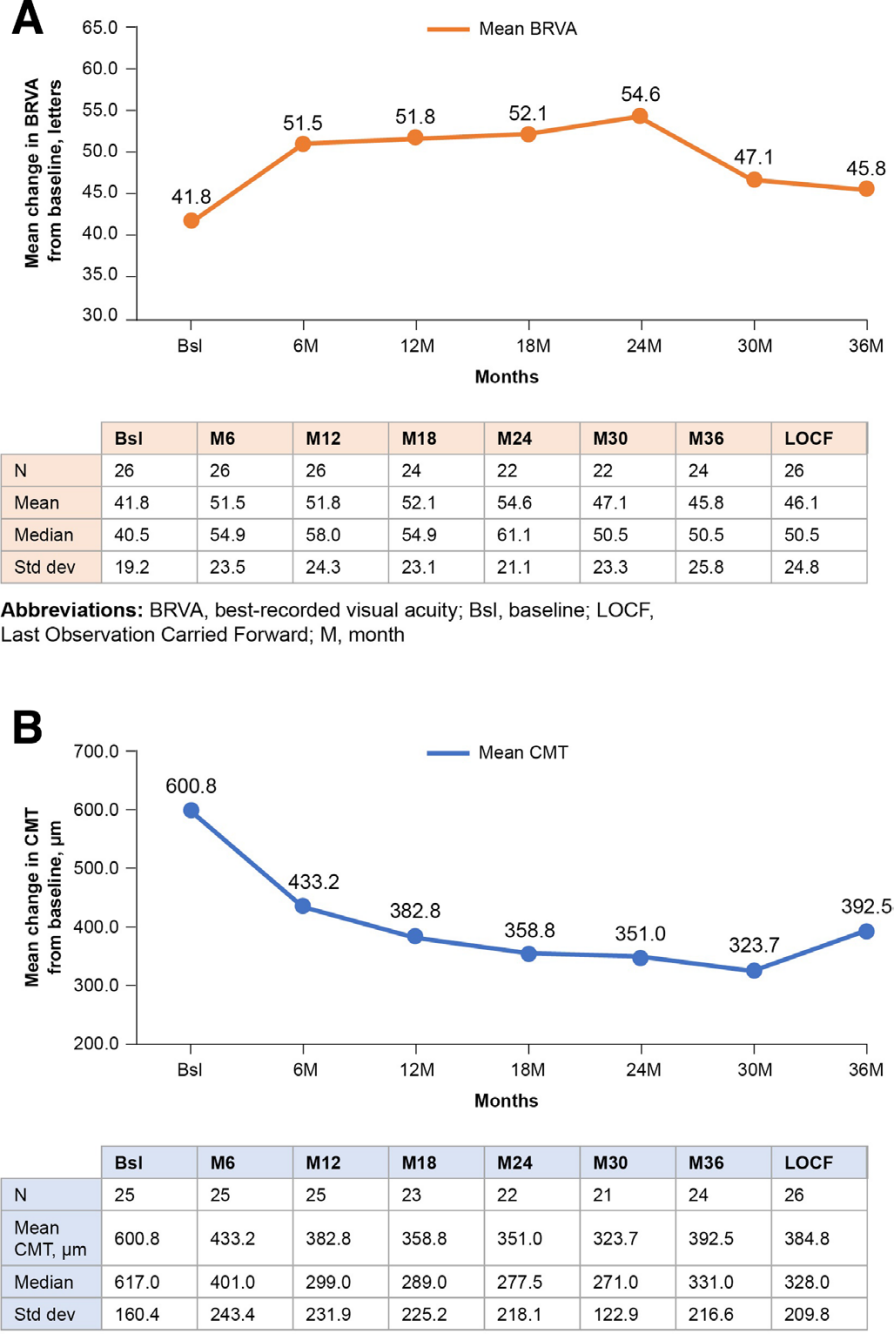
Mean change in BRVA (A) and CMT (B) from baseline over 36 months’ follow-up in all eyes post-FAc implantation. Statistically significant differences (p≤0.05, Student’s paired t-test) were observed at months 6 and 24 for both BRVA and CMT. At 36 months the reduction in CMT was sustained (p≤0.05). BRVA, best-recorded visual acuity; Bsl, baseline; CMT, central macular thickness; FAc, fluocinolone acetonide; LOCF, last observation carried forward; M, month.

Baseline BRVA was 41.8 letters (figure 1A), and following FAc implantation there was statistical improvement (p≤0.05) in mean BRVA at month 6 (51.5 letters) and month 24 (54.6 letters), and a numerical improvement at month 36 (45.8 letters; p>0.05). A decline was noted at months 30 and 36 due to loss in VA in some eyes that in the opinion of the authors was not related to the FAc implant. However, there was still a numerical improvement of 5.3 letters at month 30 (47.1 letters) and 4 letters at month 36 (45.8 letters) compared with baseline (41.8 letters).

Two eyes lost 35 letters by the end of the study, which was not regarded to be related to the FAc implant. Excluding these two eyes, the mean gain in BRVA at the last observation was 7.6 letters from a baseline of 40.9 letters. One eye lost vision due to loss to follow-up (see the IOP-related events section) and another eye developed a rhegmatogenous retinal detachment 24 months after the FAc implant had been administered. In this case the eye had been vitrectomised and received panretinal laser photocoagulation for proliferative DR 4 years earlier; DR was quiescent and adequately treated at the time the FAc implant was administered.

#### Central macular thickness

Figure 1B shows the mean change in CMT from baseline to 36 months’ follow-up. A statistically significant reduction (p≤0.05) in mean CMT was observed from 600.8 µm at baseline to 433.2 µm at month 6, 351.0 µm at month 24, and 392.5 µm at month 36.

#### VA responders

A large percentage of patients experienced clinically meaningful VA gains post-FAc implant over the follow-up period of 36 months. The percentage of eyes with vision stability/improvement was 86.4% at month 24 and 76.9% at last observation.

**Figure 2:**
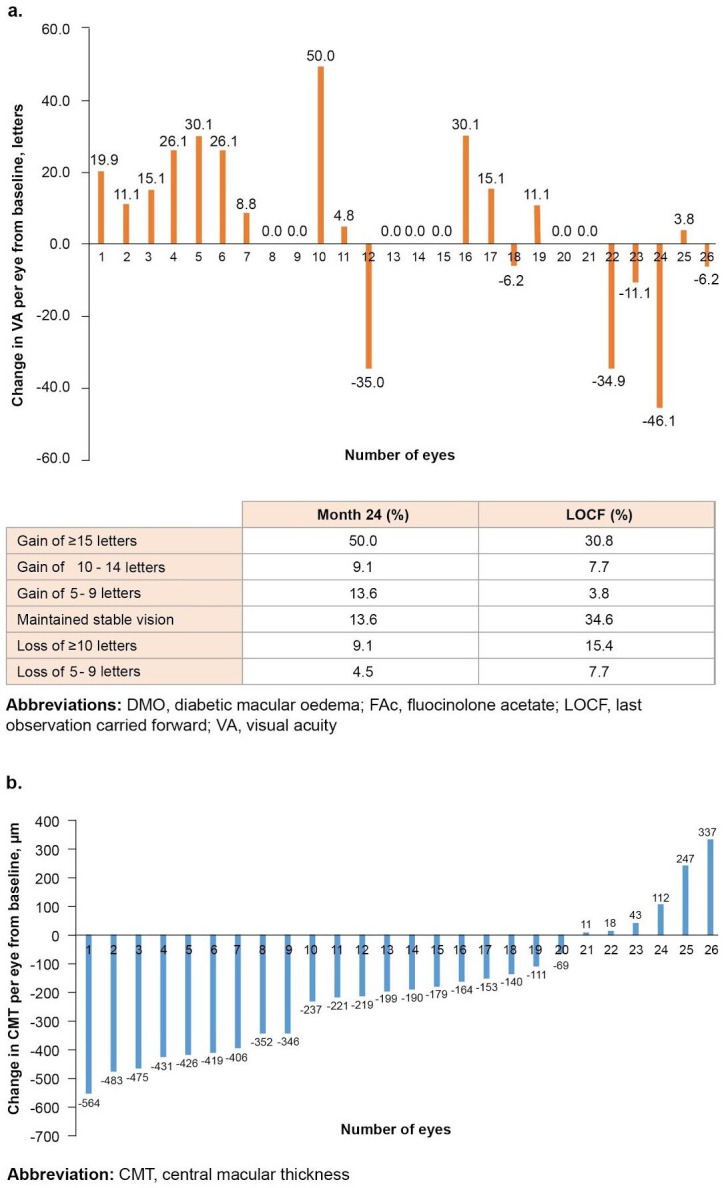
Change in VA (A) and CMT (B) per eye over 24 months from baseline following FAc implant in patients with DMO. CMT, central macular thickness; DMO, diabetic macular oedema; FAc, fluocinolone acetate; LOCF, last observation carried forward; VA, visual acuity.

Overall, 50.0% and 30.8% of eyes gained ≥15 letters at month 24 and last observation, respectively (figure 2A).

#### Anatomical responders

In the overall group, six (23.1%) patients showed no anatomical improvement in CMT (no reduction or improvement of CMT from baseline) (figure 2B). An analysis of eyes achieving a ≥20% reduction in CMT post-FAc implant revealed that 69.2% (n=18/26) experienced a ≥20% reduction in CMT post-FAc implant at month 12, with 17 of these 18 eyes sustaining this reduction through to month 36.

#### Subgroup analyses (≥20% CMT reduction at month 12)

**Figure 3:**
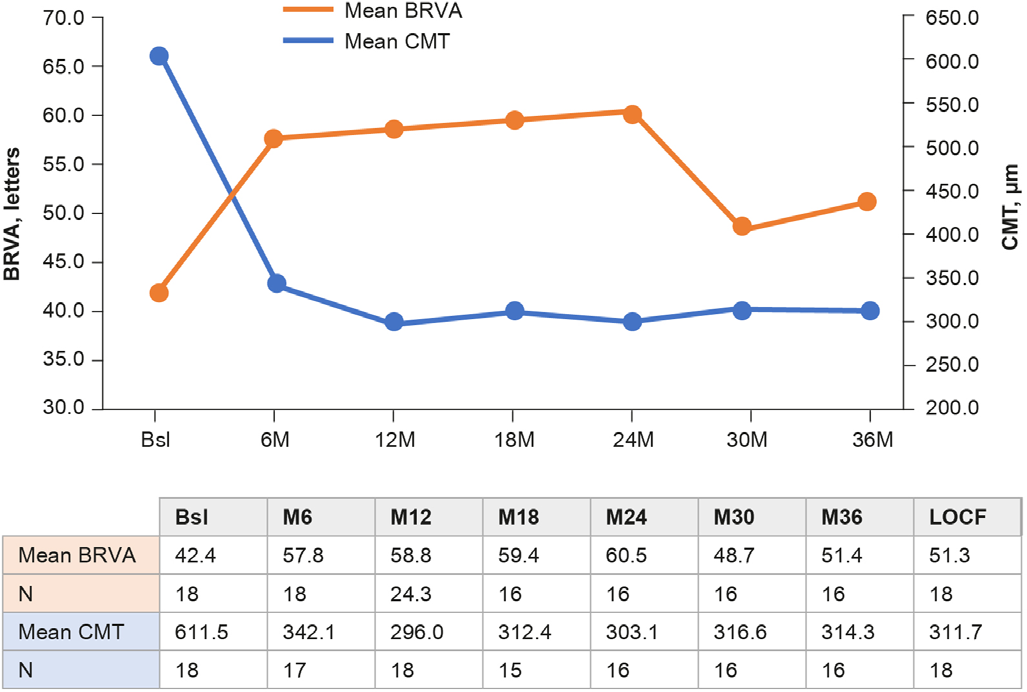
Mean change in BRVA and CMT from baseline to 3 years’ follow-up in a subgroup of patients with >20% CMT reduction at month 12 following FAc implant in patients with DMO. BRVA, best-recorded visual acuity; Bsl, baseline; CMT, central macular thickness; DMO, diabetic macular oedema; FAc, fluocinolone acetate; LOCF, last observation carried forward; M, month.

In this subgroup, the mean BRVA increased to 58.8 letters at month 12 (16.4 letter gain), 60.5 letters at month 24 (18.1 letter gain), 51.4 letters at month 36 (9 letter gain), and 51.3 letters at last observation (8.9 letter gain) from a baseline of 42.4 letters (figure 3). Marked reductions in mean CMT were observed at months 12 (296.0 µm), 24 (303.1 µm), 36 (314.3 µm) and at last observation (311.7 µm) from a baseline of 611.5 µm. Within a year post-FAc implant, BRVA and CMT improved by 16.4 letters and 315.5 µm, respectively (figure 3).

#### Treatment burden pre-FAc and post-FAc implant

Fewer treatments were required following FAc therapy, with 0.9 treatments per eye per year post-FAc vs 3.7 pre-FAc implant. A mean of one treatment every 13.33 months post-FAc implant was reported compared with a mean of one treatment every 3.24 months pre-FAc implant. At month 36, 462 hospital visits were reported post-FAc implant vs 570 hospital visits in the 36 months pre-FAc implant for all 26 eyes (p≤0.05).

### Safety

#### IOP-related events

Two of 26 eyes had an IOP elevation at baseline (readings of 25 and 27 mm Hg). One of these eyes underwent a trabeculectomy between months 24 and 30 after receiving the FAc implant. The patient had received 17 intravitreal treatments pre-FAc (including one corticosteroid) and 15 post-FAc implant and, given the IOP issues, had good VA outcome, gaining 15 letters from baseline levels.

Twelve (46.2%) eyes received IOP-lowering treatment for IOP elevation during the follow-up period. Eleven (42.3%) eyes had an increased IOP of ≥10 mm Hg and 12 (46.2%) had an increased IOP to ≥25 mm Hg. One patient was lost to IOP follow-up. In this patient, despite good initial functional response, there was a loss of 35 letters from baseline, which was thought to be due to the untreated glaucoma. This highlights the need for quarterly IOP checks to be built into clinical protocols, as defined in the Summary of Product Characteristics.

In order to illustrate the overall findings, two patient cases are described more in detail, with interventions and outcomes reported for 36 months pre-FAc and post-FAc implant.

## Cases illustrating the use of FAc implant in patients with DMO

### Patient population

#### Patient 1

**Figure 4:**
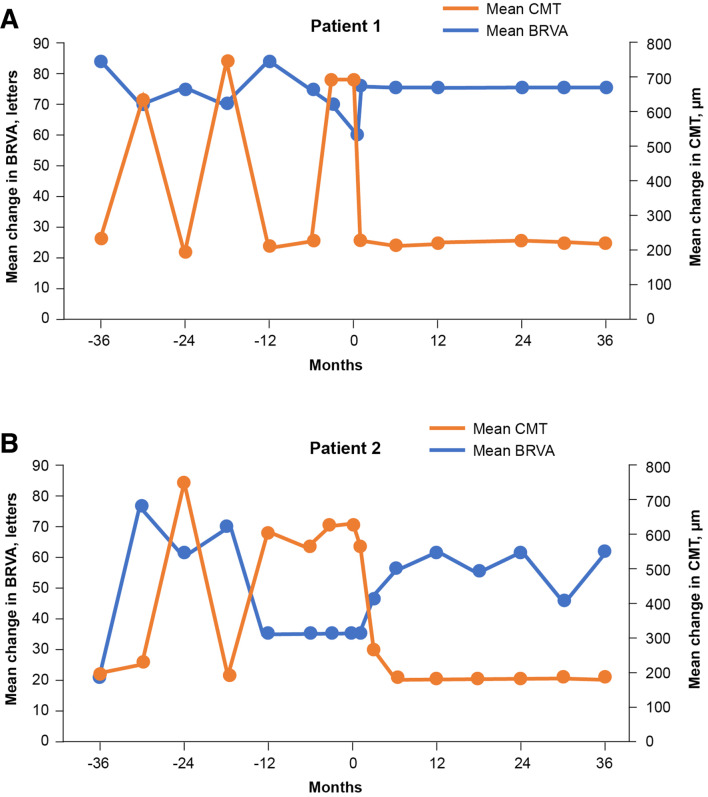
Mean change in BRVA and CMT from baseline at 1, 2 and 3 years’ follow-up in patient 1 (A) and patient 2 (B) post-FAc implantation. BRVA, best-recorded visual acuity; CMT, central macular thickness; FAc, fluocinolone acetate.

This patient was a 46-year-old man with a history of type 1 diabetes. At baseline, BRVA was 61.1 letters and CMT was 699 µm. IOP was 14 mm Hg. Prior to FAc implant, the left eye of this patient received treatment with anti-VEGF (six injections) and corticosteroid (dexamethasone, triamcinolone, two injections). Intravitreal injection of the FAc implant was conducted in the left eye on 30 October 2014 (figure 4A).

#### Patient 2

This patient was a 58-year-old man with a history of type 2 diabetes. At baseline, BRVA was 35 letters and CMT was 620 µm. IOP was 13 mm Hg. Prior to FAc implant, the left eye of this patient received treatment with anti-VEGF (nine injections) and corticosteroid (dexamethasone, triamcinolone, two injections). Intravitreal injection of FAc implant was conducted in the left eye on 27 May 2014 (figure 4B).

### Pre-FAc implantation

In patient 1, BRVA was ~70 letters or more (months −36 to −3) up until the baseline recording, where it had decreased to 61.1 letters prior to FAc administration. In patient 2, BRVA declined from 76.2 letters (month −30) and then markedly worsened from −12 months until baseline (35 letters), which correlated with the timing of the sustained oedema.

In both cases, the treatment burden from −36 to −12 months prior to FAc implant was relatively low (two intravitreal injections of a short-acting corticosteroid); however, 12 months prior to use of the FAc implant, anti-VEGF therapy was commenced and treatment intensity increased (6–9 intravitreal anti-VEGF injections). In patient 1, the CMT exceeded 500 µm on three occasions during the pre-FAc period (months −30, –18 and −3). A similar trend was seen in patient 2, with CMT in excess of 500 µm on four occasions in the pre-FAc period (months −24,–12, −6 and −3). Notably, in the latter case this increase was sustained during the 12 months prior to the FAc implant being given.

### Post-FAc implantation

#### Best-recorded visual acuity

In both patients, post-FAc implantation, BRVA increased early after treatment (by months 1 and 3, respectively; data not presented), and by month 6 had functionally improved to 76.2 letters in patient 1 and 54.9 letters in patient 2. In patient 1, this gain was maintained (15 letter gain) over 36 months from a baseline of 61.1 letters, while patient 2 had a gain in BRVA from a lower baseline BRVA of 35 letters to 61.1 letters by month 12 (26 letter gain), which was then maintained up to 36 months (figure 4A).

#### Central macular thickness

Following the intravitreal injection of the FAc implant, CMT reduced to <300 µm at month 1 (patient 1) and month 3 (patient 2) and this was maintained through to month 36. Considerable reduction in CMT was observed during the follow-up period, from a baseline of 699 μm to 224 μm at month 36 in patient 1 and from 620 μm to 189 μm at month 36 in patient 2 (figure 4B).

#### Safety: IOP-related events

In patient 1, IOP increased from 14 mm Hg at baseline to a peak of 21 mm Hg during the 36-month follow-up, and in patient 2 IOP peaked at 26 mm Hg from a baseline of 13 mm Hg. In patient 2, topical latanoprost was administered for IOP control.

## Discussion

This case series demonstrates the effectiveness and safety of the FAc implant in a real-world setting over a 3-year period. Data are presented on 26 eyes from 22 patients with DMO that recurred or persisted despite treatment. At month 36, a single injection of FAc implant in these patients led to clinically meaningful improvements in mean BRVA (4 ETDRS letters) and CMT (−208.3 µm) despite the severity of disease at baseline (BRVA: ~42 letters, CMT: >600 µm).

Baseline characteristics showed that patients predominantly had type 2 diabetes with well-controlled HbA1c. Unlike pivotal trials,^5 6^ prior to FAc implant, baseline characteristics also showed that patients had advanced disease; all cases were pseudophakic; had a low baseline BRVA (~42 ETDRS letters) and a thickened baseline CMT (>600 µm); and, had been treated with multiple intravitreal agents (a mean of 7.7 injections in 96% of eyes). Despite this, a single FAc treatment resulted in BRVA and CMT improvements, reported from 6 months (BRVA: +9.7 letters, CMT: −167.6 µm) and sustained for up to 36 months.

By 36 months there was a mean increase in BRVA (from 41.8 letters to 45.8 letters) with a rapid gain (to 49.5 letters) recorded at the first follow-up visit. BRVA improved to 51.5 letters at month 6 with a maximal effect at month 24 (54.6 letters), and thereafter a clinically meaningful gain being sustained through to month 36.

The two patient cases reported in this study suggest switching to an FAc implant early, that is, as soon as there is an inadequate response to previous treatment, can yield improved outcomes; patients with better VA at baseline showed rapid and sustained gains in BRVA (ie, >70 letters throughout) that last 36 months. This supports other real-world study results in which better VA outcomes were reported in patients treated with short-standing chronic DMO, as opposed to long-standing chronic DMO.^21^

FAc injection also resulted in a significant reduction in mean CMT, from 600.8 µm at baseline and remaining below 300 µm from month 12 through to month 36. Overall, these results are consistent with the results reported by other studies studying the effectiveness of the FAc implant in a real-world setting.^15–18^

Post-FAc implantation, the percentage of eyes that gained ≥15 letters from baseline to month 24 was 50%. These results are consistent with the outcomes reported in the FAME trials.^5 6^ Baseline CMT was high (600.8 µm) compared with other real-world FAc studies (ranging between 451 and 494 µm), which may reflect higher DMO disease activity in the current population.^15 16 21 22^ In a subgroup of patients, a ≥20% reduction in CMT was observed in 18 eyes at month 12 and was sustained in 17 of these eyes by month 36. This decrease was in line with the CMT reduction reported by two UK-based real-world studies,^16 23^ but almost double the reduction reported by a third UK-based real-world study.^15^

One common side effect resulting from the use of corticosteroids for the treatment of DMO is a possible rise in IOP. Prior to receiving the FAc implant, 2 of 26 eyes had an IOP >21 mm Hg at baseline (ie, 25 and 27 mm Hg) and another eye had an IOP of 21 mm Hg. During the 36 months post-FAc administration, 46.2% of treated eyes subsequently went on to receive IOP-lowering treatment. Accounting for the two eyes with prior IOP treatment, this percentage of eyes is similar to that reported in the pivotal FAME trial (ie, 38.4%).^5^

In those patients with pre-existing raised IOP and the one who underwent subsequent trabeculectomy, VA and CMT improved following FAc treatment. It is likely that IOP elevation was a pre-existing condition in these patients and that the FAc treatment was not the only contributing factor in these patients. Overall, one patient was lost to follow-up, which resulted in vision loss from glaucomatous damage. This highlights the need for quarterly IOP checks and good patient compliance.

On average, fewer injections of anti-VEGF agents and corticosteroids were required after the FAc implant was administered, therefore helping to reduce patient, clinician and clinic treatment burden. From a clinical perspective, the reduced CMT post-FAc implant correlates with the decreased treatment frequency, where the mean number of treatments per month was reduced (from one treatment every 3.24 months pre-FAc implant to one treatment every 13.33 months post-FAc implant). Furthermore, the calculation of hospital visits showed a reduction in the total number of hospital visits over 3 years, from 570 visits prior to FAc implant to 462 visits post-FAc implant. These findings are comparable with those reported in other real-world studies following treatment with the FAc implant.^12 23^

One strength of the current study is that patients were treated with FAc 0.2 µg/day in a real-world setting. In the FAME trials,^5 6^ patients with IOP >21 mm Hg or concurrent at screening with IOP-lowering treatment were excluded from the study. In the current study, treatment was not excluded based on these criteria and hence 2 of 26 eyes had an IOP elevation (>21 mm Hg) at baseline. Despite this, IOP outcomes were comparable with those observed in the FAME trials.

Limitations include the retrospective nature of the study design and a limited number of patients treated at a single centre, factors that limit the generalisation of findings across other centres.

The findings from this case series demonstrate the effectiveness and safety of the FAc 0.2 µg/day implant in a real-world clinical practice in patients with DMO that persists or recurs despite treatment. Patients treated with the FAc implant demonstrated rapid and sustained improvements in BRVA and CMT. These were accompanied by reduced treatment burden and associated clinic treatment visits.
